# A Comparative Analysis of *Sparisoma cretense* in Island Environments: Unraveling Metal Accumulation Differences in the Canary Islands (Spain, NW African Waters)

**DOI:** 10.3390/ani13243787

**Published:** 2023-12-08

**Authors:** Enrique Lozano-Bilbao, Alba Jurado-Ruzafa, José M. Lorenzo, José A. González, Arturo Hardisson, Dailos González-Weller, Soraya Paz, Carmen Rubio, Ángel J. Gutiérrez

**Affiliations:** 1 Grupo Interuniversitario de Toxicología Ambiental y Seguridad de los Alimentos y Medicamentos, Facultad de Medicina, Universidad de La Laguna (ULL), 38071 Santa Cruz de Tenerife, Spain; atorre@ull.edu.es (A.H.); dgonwel@gmail.com (D.G.-W.); spazmont@ull.edu.es (S.P.); crubio@ull.edu.es (C.R.); ajguti@ull.edu.es (Á.J.G.); 2 Grupo de Investigación en Ecología Marina Aplicada y Pesquerías (EMAP), Instituto de Investigación de Estudios Ambientales y Recursos Naturales (i-UNAT), Universidad de Las Palmas de Gran Canaria, Campus de Tafira, Las Palmas de Gran Canaria, 35017 Las Palmas, Spain; josemaria.lorenzo@ulpgc.es (J.M.L.); pepe.solea@ulpgc.es (J.A.G.); 3 Instituto Español de Oceanografía, Centro Oceanográfico de Canarias, 38180 Santa Cruz de Tenerife, Spain; alba.jurado@ieo.csic.es; 4 Servicio Público Canario de Salud, Laboratorio Central, 38006 Santa Cruz de Tenerife, Spain

**Keywords:** metal, Canary, fish, ICP-OES, season

## Abstract

**Simple Summary:**

The study delves into how environmental conditions impact metal concentrations in *Sparisoma cretense* tissues, crucial for advising the species’ health and broader implications for food security. Spanning 2022–2023, it notes variations in Al, Zn, Cd, Pb, Fe, and Cu levels across islands, with significant increases during warm seasons in 2023. The fluctuations arise from multiple factors: rising temperatures, marine activity, weather shifts, water quality, and human influences. Geological composition, marine currents, and sediment patterns also contribute. Understanding these complexities through ongoing research and surveillance is vital for conserving and managing marine ecosystems in the Canary archipelago.

**Abstract:**

This study investigates the impact of varying environmental conditions on the metal composition within the tissues of *Sparisoma cretense*, contributing to the understanding necessary to offer scientifically sound advice regarding the health status of this species. This knowledge extends beyond fishery production, encompassing implications for food security. The data span the years 2022 and 2023, encompassing both cold and warm climatic seasons. The concentrations of various metals, such as Al, Zn, Cd, Pb, Fe, and Cu, exhibited noteworthy variations across the islands, with significant increases recorded in 2023, particularly during the warm season. The intricate interplay between multiple factors shaped the availability of the analyzed elements in *S. cretense*. Factors such as rising temperatures during the warm season increased biological activity in marine ecosystems, seasonal fluctuations in weather conditions, water quality, and anthropogenic influences, all contributing to the observed variations in metal concentrations. Additionally, the geological composition of each island and the patterns of marine currents and sediment transport play pivotal roles in these differences. Comprehensive scientific research, monitoring, and environmental surveillance are essential for a holistic understanding of this variability and providing valuable insights for the conservation and management of marine ecosystems in the Canary archipelago.

## 1. Introduction

Heavy metals are chemical elements such as mercury, lead, cadmium, and copper, among others, that can naturally occur in oceans, but their presence in high concentrations due to human activities poses a serious threat to marine organisms. These metals can enter the water through industrial discharges, urban runoff, agriculture, and other contaminating sources. Once in the aquatic environment, these metals can accumulate in sediments or be absorbed by marine organisms. As these organisms are consumed by others in the food chain, heavy metals bioaccumulate, meaning that they progressively concentrate at higher levels at each trophic level. This bioaccumulation can have severe adverse effects on the health and functioning of marine organisms, causing harm to their reproduction, growth, immune system, and in some cases, even death. Moreover, heavy metals can be transferred to humans who consume contaminated marine products, posing a risk to human health. The monitoring and controlling of heavy metal levels in marine organisms are crucial to preserve the health of aquatic ecosystems and ensure food safety [1,2,3,4,5,6,7,8,9]. Marine fish act as bioindicators, aiding in the assessment of heavy metal concentrations within marine ecosystems. As the levels of heavy metals rise in water (due to industrial contamination, agricultural runoff, natural occurrences, etc.), fish, like most marine organisms, have the capacity to absorb and retain these metals in their tissues over their lifetimes [10,11,12,13,14,15]. Analysis of metal accumulation in fish provides scientists with valuable insights into water quality and the overall well-being of marine ecosystems. Moreover, these data are crucial in evaluating potential health risks to humans, as heavy metals can accumulate in fish that eventually become part of our diet. Consequently, monitoring marine fish plays an indispensable role in the supervision and preservation of the health of marine ecosystems and food safety [16,17,18,19].

In this context, the Canary Islands represent an interesting study case due to the uniqueness of this marine ecosystem. Positioned in the central eastern Atlantic, the oligotrophic waters surrounding these islands support a quite high marine biodiversity and play a fundamental role in the local economy, particularly for artisanal fishing and tourism [20,21,22,23,24]. Nevertheless, the increasing human activity (mainly encompassing both industry and agriculture) has raised concerns regarding potential heavy metal contamination [25,26,27], with a proven anthropic impact. For example, a significant drop in the metal content of a common marine bioindicator (i.e., *Anemona sulcate*) has been found in the Canary Islands, probably linked to the radical stop of the majority of the human activities derived from the measures taken for COVID-19 restraints [28]. 

The parrotfish *Sparisoma cretense* (Linnaeus, 1758) is a demersal species living in shallow waters up to 50 m deep, along rocky shores, in temperate-subtropical seas. In the eastern Atlantic, *S. cretense* is present in coastal waters from Portugal to Senegal, including Azores, Madeira, the Canary Islands, and Cape Verde, and in the Mediterranean Sea, the species is more common in the eastern and southern coasts. Parrotfish is considered an omnivorous species, feeding on algae and small invertebrates which browse on rocks, occupying a trophic level of 2.9 ± 0.27 [29,30,31,32]. In the Canary Islands, it is one of the most appreciated and emblematic fish and constitutes the most important demersal species (in terms of landings), included as a separated stock in the framework of the Fishery Committee for the Eastern Central Atlantic (FAO/CECAF), in which the assessment has not been possible so far [33]. In the changing context among islands in the Canary archipelago, adapted management strategies to sustain healthy and sustainable fishing practices must include the potential intra-islands variability, a supposedly significant challenge for fishery management [34,35,36,37,38,39]. Therefore, investigating how different environmental conditions affect the metal composition in *S. cretense* tissues may contribute to the knowledge necessary to provide sounded scientific advice about the health status of this species, not only in terms of fishing production, but also relating food security, via analysis of the variability of the metal content (Al, Cd, Cu, Fe, Pb and Zn) in parrotfish along with its the longitudinal distribution in the Canary Islands. 

## 2. Material and Methods

**Figure 1:**
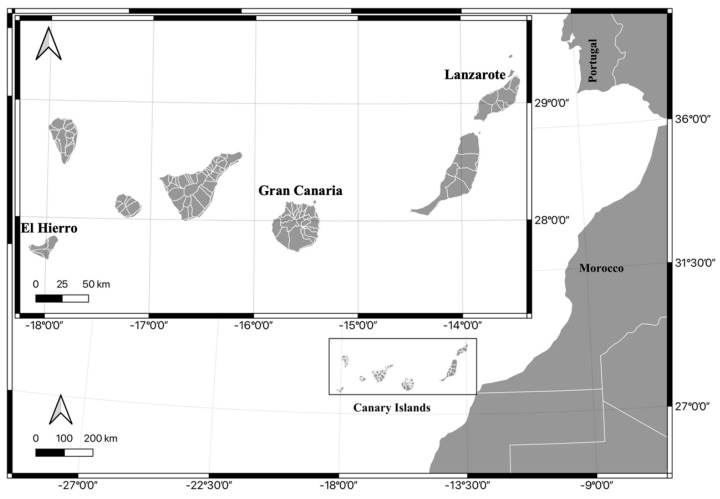
Map of the study locations.

For this study, a total of 120 *Sparisoma cretense* specimens were collected in 2022 and 2023 from commercial landings produced in the islands of Lanzarote, Gran Canaria and El Hierro, including the longitudinal range of the Canary Islands (Figure 1). The island of El Hierro was chosen because it is the island with the lowest population and tourist density and also because it is the westernmost island of the archipelago. The island of Gran Canaria is located in the center of the archipelago and has a high population and tourist density. Finally, the island of Lanzaorte is the easternmost island of the archipelago along with La Graciosa [35,40,41]. Two samplings were carried out each year, in February (the cold season) and September (the warm season). Specimens were captured employing artisanal fishing fleets specific to each of the islands with a deliberate attempt to select specimens of matching. We have avoided variation in size. Since we sampled specimens of similar sizes, we also avoided sampling with sandstorm phenomena that frequently hit the archipelago and are rich in Cu, Fe, and Zn minerals. On each island, we chose areas with similar orography, geology, and oceanographic conditions. The specimens were purchased at the fish markets of the study areas.

### 2.1. Sampling and Analysis 

Five grams of muscle tissue was extracted from the dorsal area above the pectoral fin from each sample collected. These samples were then subjected to drying in an oven at a temperature of 70 °C for 24 h. Once completely dried, they were introduced into a muffle furnace and exposed to a temperature of 450 °C ± 25 °C until they turned into white ashes. These ashes were subsequently filtered and diluted to a total volume of 25 mL, utilizing a 1.5% HNO_3_ solution. The content of Al, Cd, Cu, Fe, Pb, and Zn in the samples was quantified using Inductively Coupled Plasma-Optical Emission Spectrophotometry (ICP-OES), Thermo Scientific iCAP PRO (Waltham, MA, USA). These metals were chosen because they are anthropogenic, and all samples were above the detection limit, with a recovery of between 97 and 103.1 [42,43,44]. To assess the precision of the determinations, a quality control solution was employed after every ten samples. Furthermore, the accuracy of the analytical procedure was assessed through the analysis of the international standard reference materials DORM-1 and DORM-5, which were provided by the National Research Council of Canada. The use of these reference materials yielded a recovery rate exceeding 97%. Both the blanks and standard reference materials were analyzed alongside the samples. For accuracy, the recovery of the elements studied in the reference material was >94% in all cases. Therefore, the method used met the criteria of accuracy (established as recovery), precision (established as reproducibility), and specificity as established in the EC Regulation No. 333/2007 [45,46,47] (Table S1). The standards for the calibration curves were based on certified standard solutions. Specifically, for the metals (Al, Cd, Cu, Fe, Pb, and Zn), the SCP Science Multi-Element Std, SCP28AES certified standard was used, with a certified concentration of 100 mg/L for each of the metals. From these and for each of the metals analyzed in this study, the different concentrations of the calibration standards were prepared for the elaboration of the calibration curves, all of them in sufficient quantity for 100 mL in 1.5% nitric acid. The instrumental conditions were as follows: RF power, 1150 W; nebulizer and auxiliary gas flow, 0.5 L/min; coolant gas flow, 12.5 L/min; nebulizer gas pressure, 0.2 L/min; pump speed, 45 rpm.

### 2.2. Statistical Analysis

To assess the potential disparities in heavy and trace metal content and their relative composition in the analyzed samples, we conducted a permutational multivariate analysis of variance (PERMANOVA) using Euclidean distances. This analysis followed a 3-way design, with the fixed factors being “Island”, “Year”, and “Season”, each having varying levels. The “Island” factor had three levels (Lanzarote, Gran Canaria, and El Hierro), the “Year” factor had two levels (2022 and 2023), and the "Season" factor had two levels (Cold and Warm). The metal and trace elements considered in the analysis included Al, Zn, Cd, Pb, Fe, and Cu. For the statistical tests, we performed 9999 permutations of interchangeable units and conducted post hoc comparisons to confirm the differences between the levels of significant factors (*p*-value < 0.05). To identify clusters, we employed principal coordinate analysis (PCoA), representing elements as vectors. We carried out these statistical analyses using the software packages PRIMER 7 and PERMANOVA þ v.1.0.1. [48,49].

## 3. Results

Table 1 shows the concentrations of the metals and trace elements analyzed in specimens of *S. cretense* collected from commercial landings in El Hierro, Gran Canaria, and Lanzarote (comprising a W–E distribution across the Canary Islands) in 2022 and 2023, during both climatic seasons (cold and warm). 

For Al, noticeable fluctuations in concentrations were observed across the different islands and seasons. In all islands, increases were recorded in 2023 compared to 2022, mainly for the warm season. For example, in Gran Canaria, Al concentrations increased from 0.542 mg/kg (cold, 2022) to 1.21 mg/kg (warm, 2023). Similarly, in Lanzarote, concentrations increased during the same period. 

As for Zn, concentrations also underwent significant variations, with higher levels in the warm season of 2023. In Gran Canaria, Zn increased from 0.829 mg/kg (cold, 2022) to 1.272 mg/kg (warm, 2023). Likewise, in El Hierro, Zn concentrations increased from 0.632 mg/kg to 0.692 mg/kg between 2022 and 2023 in the warm season. 

**Figure 2:**
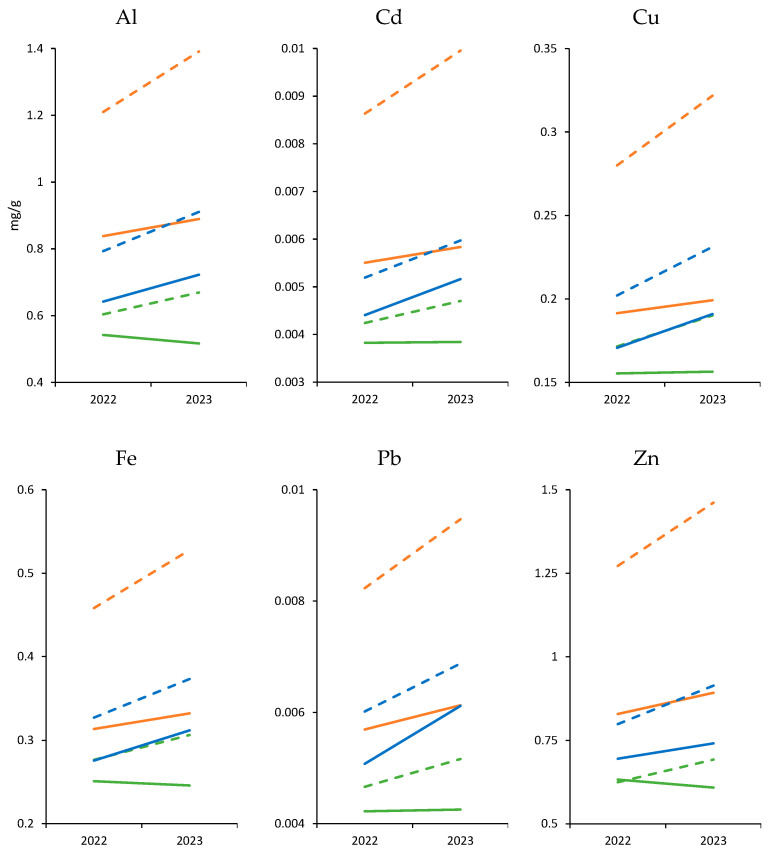
Mean line graph for each metal in mg/kg. Gran Canaria—orange; Lanzarote—blue; El Hierro—green. Continuous lines—cold seasons; doted lines—warm seasons.

For cadmium and lead, there were increases in 2023, particularly in the warm season. In Gran Canaria, Cd increased from 0.006 mg/kg (cold, 2022) to 0.009 mg/kg (warm, 2023). Similarly, in Lanzarote, the Pb concentration increased in the warm season of 2023 (Figure 2).

Regarding the iron (Fe) concentrations, they were notably higher in Gran Canaria compared to El Hierro and Lanzarote, with higher values in the warm season of 2023.

Finally, copper (Cu) also exhibited a similar trend, with higher concentrations in Gran Canaria, reaching up to 0.28 mg/kg in the warm season of 2023.

Table 2 presents a summary of the results of the three-factor analysis of variance (ANOVA), revealing significant disparities in metal concentrations across islands, years and climatic seasons. Overall, substantial variations were noted in the levels of Al, Zn, Cd, Pb, Fe, and Cu when making various comparisons. In fact, significant differences were observed among islands concerning the concentrations of Al, Zn, Cd, Pb, Fe, and Cu. Furthermore, disparities in the concentrations of certain metals seem linked to the climatic seasons (cold and warm).

The three-factor analysis of variance (ANOVA) revealed significant disparities in metal concentrations between the years 2022 and 2023 across the islands of Lanzarote, Gran Canaria, and El Hierro, as well as in the cold and warm seasons (Table 3). In general, notable distinctions were observed in the levels of Al in Lanzarote during both seasons and in Gran Canaria during the cold season. Moreover, significant variations were noted in the concentrations of Zn in Lanzarote during the cold season and in El Hierro during the warm season. However, for Cd, Pb, Fe, and Cu, most of the comparisons did not reveal significant differences.

The three-factor analysis of variance (ANOVA) unveiled noteworthy variations in metal concentrations between the cold and warm climatic seasons, considering the islands of Lanzarote, Gran Canaria, and El Hierro, along with the years 2022 and 2023 (Table 4). The p-values denote statistical significance, and in the majority of the comparisons, significant differences (*p* < 0.05) were observed. Specifically, during the cold seasons, substantial disparities were evident in the concentrations of the elements analyzed across various islands and year combinations. In the warm seasons, significant differences were mainly detected in the concentrations of Al, Zn, Pb, Fe, and Cu. Nevertheless, significant differences were not found for some comparisons, such as between the concentrations of Al in El Hierro during both seasons in 2023, and the concentrations of Cd in Gran Canaria during both seasons in 2022.

## 4. Discussion 

Since parrotfish *S. cretense* is a browser omnivorous species, its metal concentrations are closely linked to the pollutants released into the marine coastal environment, and primarily absorption by lower trophic levels significantly depends on the elemental availability in the water mass and substrate. The metal concentrations found in the parrotfish in the Canary Islands show an overall increase during the warm season that may be ascribed to a network of interconnected factors, underscoring the intricate interplay of multiple factors shaping the availability of the analyzed elements, which is not addressable with the information obtained by the present research [50,51,52]. Firstly, the rise in temperatures during the warm season may exert an influence on the presence of metals in the aquatic environment. Elevated water temperatures typically enhance the solubility and mobility of metals in the water, potentially resulting in an increased uptake by marine organisms, including *S. cretense*. Moreover, an increase in biological activity is usually observed in marine ecosystems during the warm season [53,54,55,56]. This surge could encompass increased food consumption and metabolic processes in fish, which could lead to a greater accumulation of metals by consuming prey that could contain metals in their tissues or by ingesting particles suspended in water. In addition, seasonal fluctuations in weather conditions and water quality can also impact the metal concentrations in the available food for *S. cretense*. Changes in precipitation patterns, runoff from rainwater, erosion processes, etc., can affect the amount of elements transported into bodies of water and, eventually, the marine food chain [57,58,59].

The fluctuations in metal concentrations within the *S. cretense* species across the Canary Islands (i.e., Lanzarote, Gran Canaria, and El Hierro) are ascribed to an intricate interplay of environmental and anthropogenic factors. In the case of Gran Canaria, which is characterized by a high population density and bustling tourism sector, more pronounced human influence would be expected compared to the other islands. This intense human activity significantly contributes to the variability in metal concentrations [41,60,61,62,63]. The tourism sector is leading to an expansion of coastal infrastructure and increased maritime traffic, frequently resulting in an increase in the metallic pollutants released into the marine environment. Furthermore, the population density and industrial activities in Gran Canaria probably exacerbate terrestrial pollution sources, consequently impacting coastal waters and marine ecosystems. Nevertheless, variations in the geology and physical attributes of the islands also play a pivotal role in shaping the differences in metal concentrations found in marine organisms [28,64,65,66,67]. On the one hand, the geological composition of each island influences the marine substrate and, in turn, the metal availability in the aquatic environment. On the other hand, geologic processes, such as erosion and leaching, may liberate metals into the water, affecting marine organisms [68,69,70,71]. Additionally, the patterns of marine currents and sediment transport contribute to the dispersal of metals along the coastline and among the islands [22,72]. 

Finally, the metal concentration differences observed among *S. cretense* in Lanzarote, Gran Canaria, and El Hierro occur because of a multifaceted interplay between natural variables and human activities. This intricacy underscores the significance of comprehending the consequences of pollution in marine ecosystems in this particular and unique region. In order to provide scientific advice for the conservation and management of marine ecosystems within the Canary archipelago, further scientific research, surveillance, and environmental monitoring are indispensable for a comprehensive assessment of this variability [73,74,75,76].

The use of the fish species *S. cretense* as a bioindicator of heavy metal pollution in the Canary archipelago holds significant scientific and environmental relevance. *S. cretense*, commonly known as parrotfish or vieja, has emerged as a crucial biological indicator to assess the presence and impact of heavy metals in the marine ecosystems of the Canary Islands. These fish, due to their trophic position and feeding habits, act as accumulators of contaminants, reflecting the environmental quality of their habitats. Heavy metals such as mercury, lead, cadmium, and copper, among others, pose significant concerns due to their persistence and toxicity in aquatic environments [10,30,77,78]. Analyzing concentrations of heavy metals in *S. cretense* provides valuable insights into pollution and its potential impacts on the marine ecosystems of the Canary Islands. Given its widespread distribution across different marine habitats of the islands, this species offers the opportunity to study the spatial and temporal variability in heavy metal contamination in the region [10,79,80]. The process of bioaccumulation of heavy metals in *S. cretense* is fundamental for understanding pollution dynamics in the marine food chain. These fish can accumulate heavy metals in their tissues over time through the ingestion of contaminated food and direct exposure to polluted water. This progressive accumulation of heavy metals in the tissues of *S. cretense* allows for the assessment of chronic exposure of marine organisms to pollution and, therefore, the estimation of potential impacts on the health of the biological community and potentially human health [81,82,83,84]. The selection of *S. cretense* as a bioindicator is based on its ability to reflect the variability in heavy metal levels within different marine environments of the Canary Islands. Comparing concentrations of heavy metals in this species among different locations and over time provides valuable information on pollution sources and patterns in the region.

The present study aims to analyze the concentrations of lead and cadmium in *S. cretense* fish, in order to assess food safety and compliance with the limits established by current regulations. The results obtained reveal that the concentrations of lead and cadmium in *S. cretense* samples are below the maximum values allowed according to the established regulations. These findings indicate that the consumption of this specific fish within our study area does not pose a significant risk in terms of exposure to these heavy metals, thus providing a solid scientific basis to support its food safety and promote responsible consumption within established regulatory parameters [85,86,87,88].

## 5. Conclusions

The study of metal concentrations in the parrotfish *S. cretense* in the Canary Islands highlights a complex relationship between natural ecological dynamics and anthropogenic influences in the marine coastal environment. The findings reveal a notable increase in metal concentrations during the warm season, a trend that appears to be influenced by various interconnected factors. Firstly, the rise in temperatures during the warm season plays a role in the presence of metals in the aquatic environment. Elevated temperatures enhance metal solubility and mobility in water, potentially leading to an increased uptake by marine organisms like *S. cretense*. Moreover, heightened biological activity in marine ecosystems during this period could result in greater metal accumulation through increased food consumption and metabolic processes in fish.

Seasonal fluctuations in weather conditions, water quality, and precipitation patterns also impact the availability of elements in the marine food chain. Changes in runoff from rainwater, erosion processes, and other environmental factors affect the amount of elements transported into bodies of water, influencing metal concentrations in the marine environment.

The variations in metal concentrations among the Canary Islands—Lanzarote, Gran Canaria, and El Hierro—stem from a combination of environmental and anthropogenic factors. Gran Canaria, with its high population density and bustling tourism sector, exhibits more pronounced human influence, significantly contributing to the variability in metal concentrations. The expansion of coastal infrastructure and increased maritime traffic associated with tourism often results in a higher release of metallic pollutants into marine environments. Moreover, population density and industrial activities in Gran Canaria contribute to terrestrial pollution sources affecting coastal waters.

However, the geological and physical attributes of the islands also play a pivotal role in the metal concentrations found in marine organisms. Geological composition influences marine substrate and metal availability, while geologic processes like erosion and leaching can release metals into the water. Marine currents and sediment transport patterns further disperse metals along the coastline and among the islands.

The observed differences in metal concentrations among Lanzarote, Gran Canaria, and El Hierro reflect the complex interplay between natural variables and human activities. Understanding the consequences of pollution in marine ecosystems in this unique region is crucial. Comprehensive assessment through further scientific research, surveillance, and environmental monitoring is essential for providing scientific guidance for the conservation and management of marine ecosystems within the Canary archipelago.

The use of *S. cretense* as a bioindicator of heavy metal pollution in the Canary archipelago is essential for the evaluation and understanding of the environmental quality of marine ecosystems. This scientific approach offers key perspectives for the environmental monitoring and effective management of ocean health in this unique and biodiverse region.

The monitoring of this species should continue in the same locations and be increased to one or two more islands. We will also proceed to analyze samples from Morocco and the Mediterranean, Spain, and Greece in future studies.

## Figures and Tables

**Table 1:** Descriptive statistics of the metal content in mg/kg.

	El Hierro				Gran Canaria				Lanzarote			
	2022		2023		2022		2023		2022		2023	
	Cold	Warm	Cold	Warm	Cold	Warm	Cold	Warm	Cold	Warm	Cold	Warm
**Al**	0.542 ± 0.114 (0.372–0.688)	0.604 ± 0.153 (0.391–0.825)	0.516 ± 0.102 (0.38–0.682)	0.669 ± 0.169 (0.434–0.914)	0.838 ± 0.147 (0.668–1.135)	1.210 ± 0.112 (1.029–1.398)	0.889 ± 0.145 (0.744–1.192)	1.391 ± 0.153 (1.131–1.676)	0.642 ± 0.108 (0.409–0.756)	0.793 ± 0.06 (0.674–0.86)	0.722 ± 0.072 (0.634–0.832)	0.911 ± 0.074 (0.808–1.026)
**Zn**	0.632 ± 0.14 (0.458–0.9)	0.625 ± 0.166 (0.346–0.87)	0.609 ± 0.097 (0.454–0.719)	0.692 ± 0.184 (0.383–0.964)	0.829 ± 0.159 (0.645–1.109)	1.272 ± 0.153 (1.01–1.485)	0.893 ± 0.151 (0.678–1.164)	1.462 ± 0.187 (1.11–1.636)	0.695 ± 0.154 (0.503–0.989)	0.798 ± 0.151 (0.569–1.017)	0.741 ± 0.139 (0.554–0.948)	0.914 ± 0.157 (0.683–1.118)
**Cd**	0.004 ± 0.001 (0.003–0.005)	0.004 ± 0.001 (0.003–0.005)	0.004 ± 0.001 (0.003–0.005)	0.005 ± 0.001 (0.003–0.006)	0.006 ± 0.002 (0.004–0.009)	0.009 ± 0.001 (0.007–0.011)	0.006 ± 0.002 (0.004–0.009)	0.01 ± 0.002 (0.008–0.013)	0.004 ± 0.001 (0.003–0.006)	0.005 ± 0.001 (0.004–0.007)	0.005 ± 0.001 (0.004–0.006)	0.006 ± 0.001 (0.004–0.008)
**Pb**	0.004 ± 0.001 (0.004–0.005)	0.005 ± 0.001 (0.004–0.005)	0.004 ± 0.001 (0.004–0.005)	0.005 ± 0.001 (0.004–0.006)	0.006 ± 0.001 (0.005–0.009)	0.008 ± 0.002 (0.006–0.011)	0.006 ± 0.001 (0.005–0.009)	0.009 ± 0.002 (0.007–0.013)	0.005 ± 0.001 (0.004–0.008)	0.006 ± 0.002 (0.005–0.01)	0.006 ± 0.002 (0.005–0.009)	0.007 ± 0.002 (0.006–0.011)
**Fe**	0.251 ± 0.043 (0.198–0.326)	0.277 ± 0.042 (0.224–0.343)	0.246 ± 0.045 (0.196–0.333)	0.306 ± 0.047 (0.248–0.38)	0.314 ± 0.05 (0.244–0.402)	0.458 ± 0.085 (0.365–0.619)	0.332 ± 0.041 (0.295–0.41)	0.528 ± 0.11 (0.413–0.742)	0.276 ± 0.047 (0.217–0.358)	0.327 ± 0.066 (0.246–0.441)	0.312 ± 0.039 (0.264–0.394)	0.374 ± 0.063 (0.295–0.485)
**Cu**	0.155 ± 0.027 (0.126–0.199)	0.172 ± 0.03 (0.138–0.215)	0.156 ± 0.028 (0.125–0.203)	0.19 ± 0.033 (0.152–0.238)	0.191 ± 0.033 (0.155–0.245)	0.28 ± 0.056 (0.199–0.377)	0.199 ± 0.032 (0.166–0.25)	0.322 ± 0.067 (0.238–0.452)	0.171 ± 0.029 (0.139–0.218)	0.202 ± 0.039 (0.157–0.269)	0.191 ± 0.027 (0.169–0.24)	0.231 ± 0.04 (0.188–0.295)

**Table 2:** Results of pairwise tests examining the significant factor “Year” and “Season”: a three-way ANOVA; * *p* < 0.05.

		2022		2023	
		Cold	Warm	Cold	Warm
**Al**	Lanzarote vs. Gran Canaria	0.003 *	0.001 *	0.004 *	0.001 *
	Lanzarote vs. El Hierro	0.120 *	0.004 *	0.001 *	0.001 *
	Gran Canaria vs. El Hierro	0.002 *	0.001 *	0.001 *	0.001 *
**Zn**	Lanzarote vs. Gran Canaria	0.097	0.007 *	0.067	0.001 *
	Lanzarote vs. El Hierro	0.390	0.051	0.052	0.001 *
	Gran Canaria vs. El Hierro	0.020 *	0.001 *	0.001 *	0.001 *
**Cd**	Lanzarote vs. Gran Canaria	0.080	0.001 *	0.371	0.001 *
	Lanzarote vs. El Hierro	0.123	0.052	0.001 *	0.021 *
	Gran Canaria vs. El Hierro	0.001 *	0.001 *	0.001 *	0.001 *
**Pb**	Lanzarote vs. Gran Canaria	0.356	0.001 *	0.971	0.001 *
	Lanzarote vs. El Hierro	0.654	0.065	0.001 *	0.001 *
	Gran Canaria vs. El Hierro	0.002 *	0.001 *	0.001 *	0.001 *
**Fe**	Lanzarote vs. Gran Canaria	0.135	0.001 *	0.327	0.001 *
	Lanzarote vs. El Hierro	0.278	0.080	0.001 *	0.038 *
	Gran Canaria vs. El Hierro	0.015 *	0.001 *	0.001 *	0.001 *
**Cu**	Lanzarote vs. Gran Canaria	0.119	0.001 *	0.584	0.001 *
	Lanzarote vs. El Hierro	0.278	0.091	0.029 *	0.040 *
	Gran Canaria vs. El Hierro	0.031 *	0.001 *	0.011 *	0.001 *

**Table 3:** Results of the pairwise tests examining the significant factor “Island” and “Season”: a three-way ANOVA comparing “Years”; * *p* < 0.05.

2022 vs. 2023	Lanzarote		Gran Canaria		El Hierro	
	Cold	Warm	Cold	Warm	Cold	Warm
**Al**	0.098	0.004 *	0.467	0.070	0.673	0.444
**Zn**	0.509	0.150	0.654	0.063	0.543	0.432
**Cd**	0.213	0.651	0.551	0.099	0.761	0.129
**Pb**	0.195	0.343	0.172	0.981	0.431	0.421
**Fe**	0.121	0.152	0.451	0.170	0.801	0.206
**Cu**	0.163	0.150	0.611	0.187	0.921	0.245

**Table 4:** Results of pairwise tests examining the significant factor “Island” and “Year”: a three-way ANOVA comparing “Season”; * *p*< 0.05.

Cold vs. Warm	Lanzarote		Gran Canaria		El Hierro	
	2022	2023	2022	2023	2022	2023
**Al**	0.001 *	0.001 *	0.001 *	0.001 *	0.394	0.051
**Zn**	0.181	0.031 *	0.001 *	0.001 *	0.889	0.542
**Cd**	0.098	0.078	0.001 *	0.001 *	0.338	0.077
**Pb**	0.211	0.654	0.001 *	0.001 *	0.543	0.001 *
**Fe**	0.087	0.032 *	0.001 *	0.001 *	0.239	0.019 *
**Cu**	0.081	0.030 *	0.002 *	0.001 *	0.245	0.045 *

## Data Availability

The data presented in this study are available on request from the corresponding author.

## Supplementary Materials

The following supporting information can be downloaded at: https://www.mdpi.com/article/10.3390/ani13243787/s1, Table S1: Limits of detection and quantification. Reference [89] is cited in the Supplementary Materials.

## References

[1] Hawkes S.J. (1997). What Is a “Heavy Metal”?. J. Chem. Educ..

[2] Castro-González M.I., Méndez-Armenta M. (2008). Heavy Metals: Implications Associated to Fish Consumption. Environ. Toxicol. Pharmacol..

[3] Ali H., Khan E. (2018). Bioaccumulation of Non-Essential Hazardous Heavy Metals and Metalloids in Freshwater Fish. Risk to Human Health. Environ. Chem. Lett..

[4] Bánfalvi G. (2011). Heavy Metals, Trace Elements and Their Cellular Effects. Cell. Eff. Heavy Met..

[5] Ali H., Khan E., Ilahi I. (2019). Environmental Chemistry and Ecotoxicology of Hazardous Heavy Metals: Environmental Persistence, Toxicity, and Bioaccumulation. J. Chem..

[6] Khristoforova N.K., Emelyanov A.A., Efimov A.V. (2018). Bioindication of Heavy-Metal Pollution in the Coastal Marine Waters of Russky Island (Peter the Great Bay, Sea of Japan). Russ. J. Mar. Biol..

[7] Li H., Ji H., Shi C., Gao Y., Zhang Y., Xu X., Ding H., Tang L., Xing Y. (2017). Distribution of Heavy Metals and Metalloids in Bulk and Particle Size Fractions of Soils from Coal-Mine Brownfield and Implications on Human Health. Chemosphere.

[8] Mohan S.V., Nithila P., Reddy S.J. (1996). Estimation of Heavy Metals in Drinking Water and Development of Heavy Metal Pollution Index. J. Environ. Sci. Health Part A.

[9] Kannan K., Yasunaga Y., Iwata H., Ichihashi H., Tanabe S., Tatsukawa R. (1995). Concentrations of Heavy Metals, Organochlorines, and Organotins in Horseshoe Crab, Tachypleus Tridentatus, from Japanese Coastal Waters. Arch. Environ. Contam. Toxicol..

[10] Thorne-Bazarra T., Lozano-Bilbao E., Hardisson A., González-Weller D., Rubio C., Paz S., Gutiérrez Á.J. (2023). Seagrass Meadows Serve as Buffers for Metal Concentrations in the Fish Species *Sparisoma Cretense* in the Canary Islands (Atlantic EC, Spain). Reg. Stud. Mar. Sci..

[11] Burger J. (2006). Bioindicators: A Review of Their Use in the Environmental Literature 1970–2005. Environ. Bioindic..

[12] Markert B.A., Breure A.M., Zechmeister H.G. (2003). Bioindicators and Biomonitors.

[13] Tokatli C. (2022). Comparisons of Diatoms and Fishes as Toxic Metal Bioindicator: A Case Study of an A-Class Wetland in Northwest Turkey under Effect of an Intensive Paddy Cultivation Stress. Environ. Sci. Pollut. Res..

[14] Plessl C., Otachi E.O., Körner W., Avenant-Oldewage A., Jirsa F. (2017). Fish as Bioindicators for Trace Element Pollution from Two Contrasting Lakes in the Eastern Rift Valley, Kenya: Spatial and Temporal Aspects. Environ. Sci. Pollut. Res..

[15] Peycheva K., Panayotova V., Stancheva R., Makedonski L., Merdzhanova A., Parrino V., Nava V., Cicero N., Fazio F. (2022). Risk Assessment of Essential and Toxic Elements in Freshwater Fish Species from Lakes near Black Sea, Bulgaria. Toxics.

[16] Azaman F., Juahir H., Yunus K., Azid A., Kamarudin M.K.A., Toriman M.E., Mustafa A.D., Amran M.A., Hasnam C.N.C., Saudi A.S.M. (2015). Heavy Metal in Fish: Analysis and Human Health-a Review. J. Teknol..

[17] Costa F., Coelho J.P., Baptista J., Martinho F., Pereira M.E., Pardal M.A. (2020). Mercury Accumulation in Fish Species along the Portuguese Coast: Are There Potential Risks to Human Health?. Mar. Pollut. Bull..

[18] Bencheikh Z., Refes W., Brito P.M., Prodocimo M.M., Gusso-Choueri P.K., Choueri R.B., de Oliveira Ribeiro C.A. (2022). Chemical Pollution Impairs the Health of Fish Species and Fishery Activities along the Algeria Coastline, Mediterranean Sea. Environ. Monit. Assess..

[19] Steinhausen S.L., Agyeman N., Turrero P., Ardura A., Garcia-Vazquez E. (2022). Heavy Metals in Fish Nearby Electronic Waste May Threaten Consumer’s Health. Examples from Accra, Ghana. Mar. Pollut. Bull..

[20] Uche-Soria M., Rodríguez-Monroy C. (2019). Solutions to Marine Pollution in Canary Islands’ Ports: Alternatives and Optimization of Energy Management. Resources.

[21] Domínguez L.M., Ferrer F.O. (2009). Aquaculture and Marine Biodiversity Boost: Case Examples from the Canary Islands. Water Resour. Manag..

[22] Barton E.D., Arístegui J., Tett P., Cantón M., García-Braun J., Hernández-León S., Nykjaer L., Almeida C., Almunia J., Ballesteros S. (1998). The Transition Zone of the Canary Current Upwelling Region. Prog. Oceanogr..

[23] Jiménez S., Sotillo B., Acosta C., Santamaría M.T.G. (2019). Deep-Sea Research Part II Seasonal Evolution of Small Pelagic Fi Sh Landings Index in Relation to Oceanographic Variables in the Canary Islands (Spain). Deep-Sea Res. Part II.

[24] Baztan J., Carrasco A., Chouinard O., Cleaud M., Gabaldon J.E., Huck T., Jaffrès L., Jorgensen B., Miguelez A., Paillard C. (2014). Protected Areas in the Atlantic Facing the Hazards of Micro-Plastic Pollution: First Diagnosis of Three Islands in the Canary Current. Mar. Pollut. Bull..

[25] Lozano-Bilbao E., Lozano G., Jiménez S., Jurado-Ruzafa A., Hardisson A., Rubio C., Weller D.-G., Paz S., Gutiérrez Á.J. (2020). Seasonal and Ontogenic Variations of Metal Content in the European Pilchard (Sardina Pilchardus) in Northwestern African Waters. Environ. Pollut..

[26] Lozano-Bilbao E., Lozano G., Jiménez S., Jurado-Ruzafa A., Hardisson A., Rubio C., Weller D.G., Paz S., Gutiérrez Á.J. (2020). Ontogenic and Seasonal Variations of Metal Content in a Small Pelagic Fish (Trachurus Picturatus) in Northwestern African Waters. Mar. Pollut. Bull..

[27] Lozano-Bilbao E., Lozano G., Jiménez S., Jurado-Ruzafa A., Hardisson A., Rubio C., Weller D.G., Paz S., Gutiérrez Á.J. (2021). Influence of Biometric and Seasonal Parameters on the Metal Content of Scomber Colias in Northwestern African Waters. Biol. Trace. Elem. Res..

[28] Lozano-Bilbao E., Delgado-Suárez I., Hardisson A., González-Weller D., Paz S., Gutiérrez Á.J. (2023). Impact of the Lockdown Period during the COVID-19 Pandemic on the Metal Content of the Anemone Anemonia Sulcata in the Canary Islands (CE Atlantic, Spain). Chemosphere.

[29] Froese R., Pauly D. (2023). Comment on “Metabolic Scaling Is the Product of Life-History Optimization”. Science.

[30] Afonso P., Morato T., Santos R.S. (2008). Spatial Patterns in Reproductive Traits of the Temperate Parrotfish *Sparisoma cretense*. Fish. Res..

[31] Afonso A., Gutiérrez Á.J., Lozano G., González-Weller D., Lozano-Bilbao E., Rubio C., Caballero J.M., Revert C., Hardisson A. (2018). Metals in Diplodus Sargus Cadenati and *Sparisoma Cretense*—A Risk Assessment for Consumers. Environ. Sci. Pollut. Res..

[32] Petrakis G., Papaconstantinou C. (1990). Biology of *Sparisoma Cretense* in the Dodecanese (Greece). J. Appl. Ichthyol..

[33] Beyahe M.H., Khallahi B., García-Isarch E., Fernández-Peralta L. (2020). Report of the FAO/CECAF Working Group on the Assessment of Demersal Resources–Subgroup North Nouakchott, Mauritania, 2–10 December 2019.

[34] Corral S., Manrique de Lara D.R. (2017). Participatory Artisanal Fisheries Management in Islands: Application to the Canary Islands (Spain). Mar. Policy.

[35] García-Romero L., Carreira-Galbán T., Rodríguez-Báez J.Á., Máyer-Suárez P., Hernández-Calvento L., Yánes-Luque A. (2023). Mapping Environmental Impacts on Coastal Tourist Areas of Oceanic Islands (Gran Canaria, Canary Islands): A Current and Future Scenarios Assessment. Remote Sens..

[36] Jennings S., Reynolds J.D., Mills S.C. (1998). Life History Correlates of Responses to Fisheries Exploitation. Proc. R. Soc. Lond. B. Biol. Sci..

[37] Lam V.W.Y., Allison E.H., Bell J.D., Blythe J., Cheung W.W.L., Frölicher T.L., Gasalla M.A., Sumaila U.R. (2020). Climate Change, Tropical Fisheries and Prospects for Sustainable Development. Nat. Rev. Earth. Environ..

[38] Clavelle T., Lester S.E., Gentry R., Froehlich H.E. (2019). Interactions and Management for the Future of Marine Aquaculture and Capture Fisheries. Fish Fish..

[39] Gutiérrez A., Lozano-Bilbao E., Gutiérrez-Fernández Á.J., Paz-Montelongo S., González-Weller D., Rubio-Armendáriz C., Niebla-Canelo D., Alejandro-Vega S., Hardisson A. (2023). Metal Levels in Serranus Atricauda and *Sparisoma Cretense* from the North-Eastern Atlantic Ocean—Contribution to Risk Assessment. Appl. Sci..

[40] López E.P., García F.C. (2006). Agrotourism, Sustainable Tourism and Ultraperipheral Areas: The Case of Canary Islands. PASOS Rev. De Tur. Y Patrim. Cult..

[41] Baute Díaz N., Simancas Cruz M.R., Padrón Fumero N., Herrera Priano F.Á., Rodríguez González P., Gutiérrez Taño D., Santana Turégano M.A., Guerra Lombardi V., García Altmann S., García González S. (2022). Tourism Observatory of the Canary Islands. Canary Islands Tourism Sustainability.

[42] Yuan Z., Luo T., Liu X., Hua H., Zhuang Y., Zhang X., Zhang L., Zhang Y., Xu W., Ren J. (2019). Tracing Anthropogenic Cadmium Emissions: From Sources to Pollution. Sci. Total Environ..

[43] Irabien M.J., Velasco F. (1999). Heavy Metals in Oka River Sediments (Urdaibai National Biosphere Reserve, Northern Spain): Lithogenic and Anthropogenic Effects. Environ. Geol..

[44] Raimundo J., Pereira P., Caetano M., Cabrita M.T., Vale C. (2011). Decrease of Zn, Cd and Pb Concentrations in Marine Fish Species over a Decade as Response to Reduction of Anthropogenic Inputs: The Example of Tagus Estuary. Mar. Pollut. Bull..

[45] Tyler G., Yvon J. (1995). ICP-OES, ICP-MS and AAS Techniques Compared. ICP Opt. Emiss. Spectrosc. Tech. Note.

[46] Elzey S., Tsai D.H., Rabb S.A., Yu L.L., Winchester M.R., Hackley V.A. (2012). Quantification of Ligand Packing Density on Gold Nanoparticles Using ICP-OES. Anal. Bioanal. Chem..

[47] Bakircioglu D., Kurtulus Y.B., Yurtsever S. (2013). Comparison of Extraction Induced by Emulsion Breaking, Ultrasonic Extraction and Wet Digestion Procedures for Determination of Metals in Edible Oil Samples in Turkey Using ICP-OES. Food Chem..

[48] Anderson M.R. (2004). The Resource for the Power Industry Professional. Proc. ASME Power.

[49] Anderson M., Braak C. (2003). Ter Permutation Tests for Multi-Factorial Analysis of Variance. J. Stat. Comput. Simul..

[50] Aguilar A., Borrell A., Pastor T. (1999). Biological Factors Affecting Variability of Persistent Pollutant Levels in Cetaceans. J. Cetacean Res. Manag..

[51] Renwick A.G. (1991). Safety Factors and Establishment of Acceptable Daily Intakes. Food Addit. Contam..

[52] Mostofa K.M.G., Liu C.-Q., Vione D., Gao K., Ogawa H. (2013). Sources, Factors, Mechanisms and Possible Solutions to Pollutants in Marine Ecosystems. Environ. Pollut..

[53] Yang T., Meng J., Jeyakumar P., Cao T., Liu Z., He T., Cao X., Chen W., Wang H. (2021). Effect of Pyrolysis Temperature on the Bioavailability of Heavy Metals in Rice Straw-Derived Biochar. Environ. Sci. Pollut. Res..

[54] Luoma S.N. (1983). Bioavailability of Trace Metals to Aquatic Organisms—A Review. Sci. Total Environ..

[55] Hooda P.S., Alloway B.J. (1993). Effects of Time and Temperature on the Bioavailability of Cd and Pb from Sludge-amended Soils. J. Soil Sci..

[56] Mebane C.A., Chowdhury M.J., De Schamphelaere K.A.C., Lofts S., Paquin P.R., Santore R.C., Wood C.M. (2020). Metal Bioavailability Models: Current Status, Lessons Learned, Considerations for Regulatory Use, and the Path Forward. Environ. Toxicol. Chem..

[57] Clearwater S.J., Farag A.M., Meyer J.S. (2002). Bioavailability and Toxicity of Dietborne Copper and Zinc to Fish. Comp. Biochem. Physiol. Part C Toxicol. Pharmacol..

[58] Watzke H.J. (1998). Impact of Processing on Bioavailability Examples of Minerals in Foods. Trends Food Sci. Technol..

[59] Guinot D., Ureña R., Pastor A., Varó I., Del Ramo J., Torreblanca A. (2012). Long-Term Effect of Temperature on Bioaccumulation of Dietary Metals and Metallothionein Induction in Sparus Aurata. Chemosphere.

[60] Sharpley R. (2020). Tourism, Sustainable Development and the Theoretical Divide: 20 Years On. J. Sustain. Tour..

[61] Ko J.T.G. (2001). Assessing Progress of Tourism Sustainability. Ann. Tour. Res..

[62] Fernández J.I.P., Rivero M.S. (2009). Measuring Tourism Sustainability: Proposal for a Composite Index. Tour. Econ..

[63] Hernández Sánchez N., Oskam J. (2022). A “New Tourism Cycle” on the Canary Islands: Scenarios for Digital Transformation and Resilience of Small and Medium Tourism Enterprises. J. Tour. Futures.

[64] Casagrandi R., Rinaldi S. (2002). A Theoretical Approach to Tourism Sustainability. Conserv. Ecol..

[65] Garín-Mun T. (2006). Inbound International Tourism to Canary Islands: A Dynamic Panel Data Model. Tour. Manag..

[66] Mikhailenko A.V., Ruban D.A., Ermolaev V.A., van Loon A.J. (2020). Cadmium Pollution in the Tourism Environment: A Literature Review. Geosciences.

[67] Bianchi R. (2004). V Tourism Restructuring and the Politics of Sustainability: A Critical View from the European Periphery (The Canary Islands). J. Sustain. Tour..

[68] Russell R.D. (1972). Evolutionary Model for Lead Isotopes in Conformable Ores and in Ocean Volcanics. Rev. Geophys..

[69] Watanabe M., Hokazono A., Handa T., Ichino T., Kuwaki N. (2006). Corrosion of Copper and Silver Plates by Volcanic Gases. Corros. Sci..

[70] Matus F., Amigo X., Kristiansen S.M. (2006). Aluminium Stabilization Controls Organic Carbon Levels in Chilean Volcanic Soils. Geoderma.

[71] Lozano-Bilbao E., Lozano G., Gutiérrez Á.J., Hardisson A., Rubio C., Paz S., Weller D.G. (2022). The Influence of the Degassing Phase of the Tagoro Submarine Volcano (Canary Islands) on the Metal Content of Three Species of Cephalopods. Mar. Pollut. Bull..

[72] Drews A., Greatbatch R.J. (2016). Atlantic Multidecadal Variability in a Model with an Improved North Atlantic Current. Geophys. Res. Lett..

[73] Pacyna E.G., Pacyna J.M., Steenhuisen F., Wilson S. (2006). Global Anthropogenic Mercury Emission Inventory for 2000. Atmos. Environ..

[74] Durrieu de Madron X., Guieu C., Sempéré R., Conan P., Cossa D., D’Ortenzio F., Estournel C., Gazeau F., Rabouille C., Stemmann L. (2011). Marine Ecosystems’ Responses to Climatic and Anthropogenic Forcings in the Mediterranean. Prog. Oceanogr..

[75] Meskhidze N., Chameides W.L., Nenes A., Chen G. (2003). Iron Mobilization in Mineral Dust: Can Anthropogenic SO_2_ Emissions Affect Ocean Productivity?. Geophys. Res. Lett..

[76] Glover A.G., Smith C.R. (2003). The Deep-Sea Floor Ecosystem: Current Status and Prospects of Anthropogenic Change by the Year 2025. Environ. Conserv..

[77] Murphy C.B., Spiegel S.J. (1983). Bioaccumulation and Toxicity of Heavy Metals and Related Trace Elements. J. Water Pollut. Control Fed..

[78] Murphy C.B. (1981). Bioaccumulation and Toxicity of Heavy Metals and Related Trace Elements. J. Water Pollut. Control Fed..

[79] Espino F., González J.A., Haroun R., Tuya F. (2015). Abundance and Biomass of the Parrotfish *Sparisoma Cretense* in Seagrass Meadows: Temporal and Spatial Differences between Seagrass Interiors and Seagrass Adjacent to Reefs. Environ. Biol. Fishes.

[80] Domingues V.S., Alexandrou M., Almada V.C., Robertson D.R., Brito A., Santos R.S., Bernardi G. (2008). Tropical Fishes in a Temperate Sea: Evolution of the Wrasse Thalassoma Pavo and the Parrotfish *Sparisoma Cretense* in the Mediterranean and the Adjacent Macaronesian and Cape Verde Archipelagos. Mar. Biol..

[81] Ramos-Miras J.J., Sanchez-Muros M.J., Morote E., Torrijos M., Gil C., Zamani-Ahmadmahmoodi R., Martin J.A.R. (2019). Potentially Toxic Elements in Commonly Consumed Fish Species from the Western Mediterranean Sea (Almería Bay): Bioaccumulation in Liver and Muscle Tissues in Relation to Biometric Parameters. Sci. Total Environ..

[82] Bordbar L., Dassenakis M., Catsiki V.A., Megalofonou P. (2015). Influence of a Ferronickel Smelting Plant Activity on the Coastal Zone through Investigation of Metal Bioaccumulation on Two Gastropod Species (Patella Caerulea and Phorcus Turbinatus). J. Environ. Anal. Toxicol..

[83] Escobar-Chicho M., Soto L.A., Vanegas-Pérez C., Estradas-Romero A. (2019). Heavy Metal Bioaccumulation in the Anemone Paraphelliactis Pabista Dunn, 1982 (Actiniaria: Hormathiidae) from the Hydrothermal System of Guaymas Basin, Gulf of California. Bull. Environ. Contam. Toxicol..

[84] Gaudry A., Zeroual S., Gaie-Levrel F., Moskura M., Boujrhal F.Z., El Moursli R.C., Guessous A., Mouradi A., Givernaud T., Delmas R. (2007). Heavy Metals Pollution of the Atlantic Marine Environment by the Moroccan Phosphate Industry, as Observed through Their Bioaccumulation in Ulva Lactuca. Water Air Soil Pollut..

[85] (2014). Reglamento (UE) No 488/2014 DE LA COMISIÓN de 12 de Mayo de 2014 Que Modifica El Reglamento (CE) No. 1881/2006 Por Lo Que Respecta al Contenido Máximo de Cadmio En Los Productos Alimenticios.

[86] (2011). Reglamento (CE) No 420/2011 de La Comisión de 29 de Abril de 2011 Que Modifica El Reglamento (CE) No 1881/2006, Por El Que Se Fija El Contenido Máximo de Determinados Contaminantes En Los Productos Alimenticios.

[87] (2006). Reglamento (CE) No 1881/2006 DE LA COMISIÓN de 19 de Diciembre de 2006 Por El Que Se Fija El Contenido Máximo de Determinados Contaminantes En Los Productos Alimenticios.

[88] (2015). Reglamento (UE) 2015/1005 DE LA COMISIÓN de 25 de Junio de 2015 Que Modifica El Reglamento (CE) No. 1881/2006 Por Lo Que Respecta al Contenido Máximo de Plomo En Determinados Productos Alimenticios.

[89] IUPAC (International Union of Pure and Applied Chemistry) (1995). Nomenclature in Evaluation of Analytical Methods including Detection and Quantificaction Capabilities. Pure Appl. Chem..

