# Supplementary material for: A Comparative Analysis of Sparisoma cretense in Island Environments: Unraveling Metal Accumulation Differences in the Canary Islands (Spain, NW African Waters)
Source: Animals (Basel). 2023 Dec 8;13(24):3787. doi: 10.3390/ani13243787 (PMC10740405; doi:10.3390/ani13243787)
Supplement: Supplementary file 1 [file animals-13-03787-s001.zip › animals-2727477-supplementary.pdf]

Supplementary

The criteria, from an analytical point of view, that guarantee the correct evaluation of the results presented in the work have been the validation, with the parameter of Accuracy, which includes the Trueness of the method (established as recovery) and the Precision of reproducibility (established as coefficient of variation) applied to the reference materials. The required trueness and coefficient of variation values were set at a maximum of 10%, obtaining lower values for all the metals under study. In addition, the Specificity (which has been carried out to verify that the method is free of spectral interferences for each of the metals studied) was also studied.
To establish the linearity of the calibration lines, the R2 value was not used, instead the use of the relative calibration errors was employed, setting as acceptance criterion a maximum of 15% of this error for all metals in the lowest standards of each line and 10% in the rest of the points of the calibration lines.
The instrumental detection and quantification limits were estimated based on the instrumental response of the equipment. Specifically, they were determined by analyzing 15 blanks under reproducibility conditions (IUPAC, 1995)1. The instrumental detection and quantification limits are shown in the following table:

Table S1. Limits of detection and quantification.

Metal	Detection Limit (LD) (mg/l)	Limit Quantification (LQ) (mg/l)
**Al (167.0 nm)**	0.005	0.015
**Cd (214.4 nm)**	0.0007	0.002
**Cu (324.7 nm)**	0.003	0.011
**Fe (238.2 nm)**	0.004	0.013
**Pb (220.3 nm)**	0.0009	0.003
**Zn (213.8 nm)**	0.0027	0.009

[1] IUPAC (International Union of Pure and Applied Chemistry). (1995). Nomenclature in Evaluation of Analytical Methods including Detection and Quantificaction Capabilities. Pure Appl Chem 67:1699-1723.
